# Home injury prevention attitude and performance: a community-based study in a designated safe community

**DOI:** 10.5249/jivr.vo112i2.1506

**Published:** 2020-07

**Authors:** Mohammad Saadati, Jafar Sadegh Tabrizi, Ramin Rezapour, Riaz Alaei Kalajahi

**Affiliations:** ^*a*^ Road Traffic Injury Research Center, Tabriz University of Medical Sciences, Tabriz, Iran.; ^*b*^ Tabriz Health Services Management Research Center, Health Management and Safety Promotion Research Institute, Tabriz University of Medical Sciences, Tabriz, Iran.; ^*c*^ Iranian Center of Excellence in Health Management, School of Management and Medical Informatics, Tabriz Universi-ty of Medical Sciences, Tabriz, Iran.; ^*d*^ Department of Health Management and Economics, School of Public Health, Tehran University of Medical Sciences, Tehran, Iran.

**Keywords:** Attitude, Performance, Home Injuries, Mothers

## Abstract

**Background:**

Unintentional injuries in the home are one of the threats to childhood quality of life which is considered as a social determinant of health. Regarding mother's leading role in taking care of the children in Iranian families, the present study was conducted to investigate mothers' home-injury prevention attitude and performance and its contributing factors in Sahand safe community, East-Azerbaijan, Iran.

**Methods:**

This was a cross-sectional study conducted in 2017. Sampling was done using "Random Sampling method" among all mothers having at least one U-5 child and attended the health centers to receive childcare services. A valid attitude questionnaire and safety performance checklist were used for data collection. Data were analyzed by SPSS-24, using descriptive (frequency, mean, etc.) and inferential statistics (chi-square, Kruskal-Wallis).

**Results:**

The mean age of mothers was 30.58 (±5.01). About 65% of the mothers had primary or secondary school education. The mean score of mothers' attitude was 72.12(±6.79). More than 58% of the mothers had an appropriate level of attitude. The mothers' injury prevention performance mean score was 66.59 (±12.85). Family’s socioeconomic status, mother's age, educational level, and job, father's job, age, and gender of the child were the contributing factors (p less than 0.05).

**Conclusions:**

Most of the mothers have an appropriate level of home-injury prevention attitude but a low level of performance. Strengthening Primary Health Care system in safe communities would have a leading role in child safety promotion through increasing the mother's knowledge, attitude and performance level.

## Introduction

Injuries are considered as one of the leading and avoidable causes of the disabilities and death in most of the countries.^1,2^ Over the recent decades, child death due to injuries have increased in contrast to the reduction in child death due to chronic and infectious diseases.^3^ Children are one of the most vulnerable groups regarding injuries. In other words, children have the highest number of injury victims relative to their population.^2^ Unintentional injuries introduced as the cause of 750000 deaths and 400 million disabilities in children annually, which impose a tremendous financial burden on the families and health system.^4-6^

Home is the place where the family and especially children spend their most time. It is a place where often some serious injuries are inflicted on children. ^7^ Unlike the general belief, considering home as a safe place; about one-third of the child's injuries like falls, burns, cuts, electric shock, asphyxia, etc. occur at home.^5^ Wiseman et al. (2002), in a study on newborn babies to 14-year-old children, concluded that 51.9% of the child injuries occurred at home, and the younger the children were with the higher frequency and number of the home injuries.^8^ According to the National Safe Kids Campaign report in the USA, 40% of the deaths and 50% of the unintentional injuries resulting in death have occurred inside or around the homes. ^9^ Literature indicated that more than 4 million children are injured annually, where most cases occur due to falls, poisoning, and burns^10^ and the most significant risks of such accidents are connected with home environments.^11^ Often, parents are adequately familiar with the risks of the injuries at home;^10^ however, they do not have enough information about the problems arising from the injuries to children,^12^ and usually they do not think about the probability of the risk of injuries, especially about their children, during the daily life interactions with their children. ^10^

Also, the results of the studies showed that the parents do not believe much in the idea that the risks resulting from the injuries can be prevented.^13,14^ This is the case, while they believe that they can provide safe conditions for their children to some extent.^10,12^ The family's socioeconomic status, education, and occupation of parents, children’s age and gender are considered as the factors affecting the attitude and safety performance of mothers in different societies.^13,15-17^

Considering the fact that the experience and quality of life during childhood has been introduced as one of the social determinants of health^18^ and regarding the outstanding role of mothers in preventing injuries in the children under 5 years old in Iranian families, the present study was conducted to investigate mothers' attitude and performance of home injuries prevention in under-5-years child in Sahand safe community, Iran.

## Methods 

**Study design and context**

This was a cross-sectional study that had been conducted in Sahand New Town from February to March 2017. Sahand is one of the new towns in East Azerbaijan Province, located in the capital city of the province in Osku County. Sahand Town is considered the 7th most populated town in East Azerbaijan Province and the most populated town in Osku County with a population of 82494 (2016 census). Safe Community program started in 2015 in Sahand, and it was designated as 407th safe community in the International Safe Community Network. The Safe Community program has been proposed by the World Health Organization (WHO) Collaborating center on community safety promotion at Karolinska Institute, Sweden, for safety promotion and injury prevention in the communities based on inter-sectoral collaboration and public participation.^19^

Sahand has three healthcare centers as Primary Health Care (PHC) facilities which each of them are in a district of city and provides health services for covered population (100%). PHC services are provided by these centers and are not deliverable from other facilities. 

**Study population**

The mothers with at least one child under five years old who have attended Sahand PHC centers were selected as the study population. The registered study population (mothers with at least one child under 5) was calculated as 6775 individuals in Sahand. Cochran formula was employed to determine the sample size, and sample size was calculated as to be 370. The population was distributed among the three healthcare centers using the proportional allocation method considering the number of the qualified individuals who were covered by each PHC centers, to cover the whole population, and the share of each center was determined (Table 1).

**Table 1:** Sample distribution in three health centers.

PHC center	Study population coverage %	Sample size
Number 1	28	104
Number 2	42	155
Number 3	30	111
Total	100	370

The sampling was done using a random sampling method such that mothers attending the centers to receive routine childcare services were invited randomly (based on random numbers) to participate in the study.

**Data collection instrument**

A standard questionnaire designed by Hatamabadi et al. (2014) was employed to study the mothers' attitudes towards the prevention of home injuries. The questionnaire had two following general sections: the demographic information including mother's age, child age, child number, family's socioeconomic status, parents' jobs and the second section including 16 questions about prevention of home injuries that were answered according to 4 point Likert scale namely strongly agree, agree, disagree, strongly disagree. The checklist of mother's safety performance at home included six groups of common home accidents, including falls, burns, drowning and asphyxia, electric shock, drug, and chemical poisoning, cuts, and traffic safety comprising 30 questions in total. 

The checklist questions were designed in a way as to be answered by the words "yes," "no," and "Not Applicable." To ensure the validity of the instrument, they were reviewed by experts, and based on their opinions some improvements were made on question texts. The members of the expert panel included the health managers (n=2), epidemiologists (n=2), family health experts (n=2), health education experts (n=1), and injury prevention experts (n=3). The validity of the attitude questionnaire and safety performance checklist were confirmed as CVI=0.96 and CVI=0.83.

First, the goal of the study was explained to the mothers; then, the questionnaires were completed through the interview by the researcher or self-reporting after obtaining their consent concerning the participation in the study.

**Data analysis**

Data analysis was conducted using SPSS 24. The state of mothers' attitudes was divided into two groups, that is, appropriate and inappropriate. The median of mothers' attitude has been employed as the basis for the division that is one of the conventional methods used for classification.^20^ Kolmogorov smirnov test showed that mothers home-injury prevention performance score distribution was not normal (p<0.05). So we have used non-parametric tests to analyze data. The data were analyzed using the descriptive statistics, including frequency, mean and standard deviation, and inferential statistics appropriate to data normality including chi-square and Kruskal–Wallis test. 

## Results

**Demographics**

Totally 370 mothers with children under five years old participated in the study. The average age of mothers was 30.58. About 65% of the mothers had high school diplomas, and lower educational degrees and 90.1% of the mothers were housekeepers. More than half of the mothers (53.6%) had only one child. Most of the families (73.7%) were considered to be middle class, in terms of economy. 45.9% of the heads of household (fathers) were self-employed. Most of the mothers (67.2%) used social security insurance. Concerning the gender of the children, 50, 9% of the children were boys, and most of whom (28.6%) were in the 4-5 year age group. Most of the families (49.7%) have only one child.

**Mothers' injury prevention attitude **

The mother's injury prevention attitude mean score was calculated to be 72.12 (±6.79) out of 100. About 58.6% of the mothers had an appropriate level of attitude towards the prevention of home injuries. Most of the mothers (69.9%) announced that they had enough ability to take care of their children. 66.9% of the mothers believed that the minor in-home injuries to children are considered to be normal. Only 14.8% of the mothers believed that the in-home injuries were not that serious. Also, 85.1% of the mothers opposed the idea that these injuries could not be avoidable. Besides, 94.1% and 94.5% of the mothers agreed that prevention of in-home injuries would result in a reduction in the waste of money and time, respectively.

Chi-square test showed that the mother's injury prevention attitude had a significant association with child age, mother educational level, age, job, family economic level and fathers' job (Table 2).

**Table 2:** Factors affecting mothers’ injury prevention attitude.

Variable name	Variable Groups	Status of Attitude		P. Value
		Inappropriate N (%)	Appropriate N (%)	
**Childs age (Month)**	0-6	48(23.4%)	23(16.8%)	<0.0001
	7-12	42(20.5%)	23(16.8%)	
	13-24	37(18%)	29(21.2%)	
	25-48	17(8.3%)	24(17.5%)	
	49-60	61(29.8%)	38(27.7%)	
	Total	205(100%)	137(100%)	
**Childs Sex**	Male	110(55.6%)	64(45.7%)	.75
	Female	88(44.4%)	76(54.3%)	
	Total	198(100%)	140(100%)	
**Mothers education status**	Diploma or lower	0	3(2.1%)	<0.0001
	Upper-diploma	135(66.5%)	90(63.8%)	
	BSc	24(11.8%)	14(9.9%)	
	MSc	40(19.7%)	31(22.2%)	
	PhD	4(%2)	3(2.1%)	
	Total	203(100%)	141(100%)	
**Fathers job status**	Employee	46(22.7%)	28(19.7%)	<0.0001
	Worker	59(29.1%)	54(28%)	
	Market	98(48.3%)	60(42.3%)	
	Total	203(100%)	142(100%)	
**Mothers job status**	Employee	13(6.4%)	10(7%)	<0.0001
	Worker	0	1(.7%)	
	Housekeeper	185(90.7%)	127(89.4%)	
	Market	6(2.9%)	4(2.8%)	
	Total	204(100%)	142(100%)	
**Family Income**	Very lower than medium	2(1%)	2(1.4%)	<0.001
	Lower than medium	43(21.1%)	33(23.2%)	
	Medium	153(75%)	103(72.5%)	
	Higher than medium	6(2.9%)	4(2.8%)	
	Total	204(100%)	142(100%)	
**Mothers age**	<25 years	32(17.8%)	23(18.5%)	<0.0001
	25–30 years	68(37.8%)	25(20.2%)	
	>30 years	80(44.4%)	76(61.3%)	
	Total	180(100%)	124(100%)	

**Mothers' injury prevention performance **

**Figure 1:**
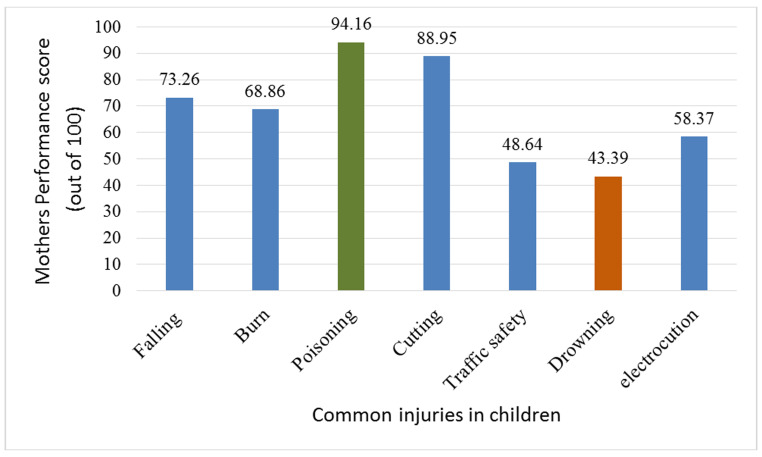
Mothers' injury prevention performance toward common injuries in children.

Mothers' injury prevention performance mean score was obtained to be 66.59 (±12.58), where the poisoning prevention got the highest score 94.15% (19.87) and drowning prevention got the least score, 22.98 (43.39%) (Figure 1).

Most of the participants' homes (90.4%) had railings or fence in the staircase. Most of the participants' homes (93.5%) were equipped with lighting systems. Also, 87.8% of the mothers pointed out that they place the hot Kettle or Samovar out of children's reach. 63.6% of mothers announced that the children use safety belts when swinging or using the baby carriage. The drugs and poisons were kept in sealed places in 94.6% of the cases. Mothers (93.8% and 95.7%) said that they kept the detergents and meat grinders out of reach of the children. Also, 70.6% of the homes were not equipped with the carbon monoxide detector in the child rooms. 51.1% of the mothers did not leave their children alone when bathing their children in the bathtubs. 52.4% of the homes were equipped with power plugs with protective cover, and 50.5% of the power plugs were placed at a high place out of reach of children. 

Mothers' educational level and age and the family's economic level have significant association with their injury prevention performance (Table 3).

**Table 3:** Factors affecting the mother’s injury prevention performance.

Variable name	Variable Groups	Total practice score		P. Value
		Mean	SD	
**Child sex**	Male	66.53	13.04	.714*
	Female	66.34	12.52	
**Child age (Month)**	0-6	63.58	16.81	.404**
	7-12	69.40	11.33	
	13-24	67.07	10.67	
	25-48	68.56	12.05	
	49-60	65.80	11.00	
**Mothers education level**	Diploma or Lower	75.00	4.30	.008**
	Upper-Diploma	65.69	11.84	
	BSc	69.91	12.61	
	MSc	66.66	16.05	
	PhD	71.66	12.84	
**Father job **	Employee	68.10	13.98	.140**
	Worker	66.60	11.61	
	Market	65.97	13.19	
**Mother job **	Employee	66.80	21.84	.143**
	Worker	73.33	0	
	Housekeeper	66.84	11.81	
	Market	60.66	16.54	
**Family Income**	Very lower than medium	60.00	23.57	.003**
	Lower than medium	64.09	9.75	
	Medium	67.16	13.33	
	Higher than medium	75.00	12.98	
**Mothers age**	<25 years	65.41	9.58	.003**
	25–30 years	69.92	11.85	
	>30 years	65.24	13.22	

* P-value was based on Mann–Whitney test. **P-value was based on Kruskal-Wallis test.

## Discussion

The results showed that about 59% of mothers had an appropriate level of attitude towards home injury prevention. Also, mothers' injury prevention performance in under-5-years old child was estimated to be 66.59 (±12.85). The poisoning prevention performance got the highest score. Also, mothers' educational level, age, job, fathers' job and economic level of families emerged as mothers' injury prevention attitude and performance predictors. 

Hatamabadi et al. (2012) study results revealed that 57% of the mothers, living in Tehran, had a positive attitude towards preventing the in-home injuries that are consistent with our study results.^17^ Bennet Murphy reported that none of the mothers participating in the study stated the injury prevention as a mothers' responsibility.^14^ Also, in the present study, 66.9% of mothers considered minor injuries to be normal; however, more than 85% of the mothers believed that in-home injuries could be prevented. This was similar to the findings of the Hooper et al. (2003) in New Zealand and the study conducted by Vincenten et al. (2005) in 14 European countries announcing that 84% and 75% of mothers believed that the in-home injuries could be avoidable, respectively.^21,22^ On the other hand, the results of studies in Canada and America reported that most mothers believed that home injuries are unavoidable.^13,14^ Regarding the fact that the mothers' attitude and performance are influenced by the community culture and the use of various methodologies in the studies, the difference in the results of studies could be discussed.

In the case of child sex, Sabely et al. (2014) reported that 58.7% of the in-home injuries occurred to boys.^23^ Similarly, the results of a study by Kamel et al. also indicated that boys were in the subject for injuries in 63% of cases.^24^ The previous literature also supported the same issue from Egypt, Turkey, and India such that more than 50% of the children injured in these studies were boys.^16,25,26^ Because the safety and health of the children under five years old mostly depend on their mothers; therefore, the mothers especially the mothers having sons should have better injury prevention knowledge, attitude, and performance. The results of our study showed that more than 55% of mothers having sons do not have an appropriate injury prevention attitude. No significant difference was observed between the mothers having sons and the mothers having daughters in injury prevention performance. Primary health care system, as the first level of health services delivery and the system with a continuous relationship with mothers, needs to promote the injury-prevention performance of the mothers as a part of their childcare services.

Hatamabadi et al. (2014) have introduced the salary of fewer than 300 dollars a month as one of the effective factors leading to low safety knowledge and attitude of mothers regarding injury prevention.^17^ Juhee Hong et al. (2010) stated the family's socioeconomic status, including the educational levels of parents, residence place, occupation and income level as a determinant factor of home injury prevention attitude and performance of mothers.^15^ In the present study, a significant association was observed between the family's socioeconomic status and mothers' injury prevention performance and attitude. Job and educational level of the mother had a significant association with her attitude and performance in employing injury prevention strategies. Also, Sabley et al. (2014) had pointed out that mothers having higher educational levels have higher knowledge about the ways to prevent in-home injuries than mothers with lower education.^23^ Similarly, the significant association between mothers' education and better home injury performance was pointed out in the study by Kamel et al.^24^ However, according to the results of the study conducted by Lafta et al. (2014) in Iraq revealed some contrary results to the findings of the present and the previous studies, that mothers who possess higher educational levels, had a lower level of knowledge about home injuries prevention.^27^ Households' socioeconomic status is a function of community macro-policies. Therefore policy-makers should be aware of their policies effects not only on child safety but also on families' general health. Improving building standards towards building child-friendly homes might be a good policy initiative to promote childhood quality in communities implementing safe community program. 

Generally, it is expected that the attitude of individuals affect their behavior, but the lack of a significant association between the injury prevention attitude and mother’s performance was one of the most noticeable results of the study. In other words, the high levels of mothers' home injury prevention attitude would not necessarily result in better performance. This issue has been pointed out in previous studies that mothers high injury prevention knowledge leads to the high level of attitude; however, the high level of their knowledge and attitude would not result in better performance or behavior in the prevention of the injuries.^17^ This finding shows the necessity of paying attention to the change in behavior and creating new behavior patterns resulting from the education provided for the mothers by PHC system. Interventions to improve child safety need strong cross-sectional collaboration which is emphasized in safe community program.^28^

It should be considered that childhood quality, as one of the social determinants of health, can manifest throughout the lifetime of individuals. The important point is the adoption of proper educational and cultural policies to make changes in mothers' safety behavior to promote children's quality of life.^29^ This would lead to a decrease in the injuries costs imposed on the families and the healthcare system. Using the mass media like TV, the radio and social networks to enhance mothers' knowledge and attitude, safety education to children at homes and kindergartens through drawing, animations etc. specially for the boys and the necessity to employ the safe equipment in leisure places and building houses are considered the items that can be taken into consideration in this regard.

## Conclusion

The results showed that more than 58% of the mothers had an appropriate level of home-injury prevention attitude. However, the performance of mothers was not at an acceptable level. Mother age, education level, parents' jobs, family's socioeconomic status, child age, and gender were considered as contributing factors to mothers' injury prevention attitude and performance. Deprived residency areas should be considered for higher support to prevent injuries. Strengthening the PHC system role in safe community programs could have a significant effects in child safety promotion through mothers' knowledge, attitude and performance (KAP) promotion.

**Limitations**

Paternalistic factors were not addressed much. Of course, due to the critical role of mothers in childcare comparing to fathers, in Iranian families, this might not have a considerable effect on results.

**Abbreviations**

WHO: World Health Organization 

**Availability of data and material**

The datasets used and analyzed during the current study are available from the corresponding author on reasonable request.

**Acknowledgments**

We appreciate the kind collaboration of the managers and personnel of Sahand health centers and mothers participating in the study.
